# 1-[2-(2,6-Dichloro­benz­yloxy)-2-(2-fur­yl)eth­yl]-1*H*-benzimidazole

**DOI:** 10.1107/S1600536808020758

**Published:** 2008-07-09

**Authors:** Özden Özel Güven, Taner Erdoğan, Simon J. Coles, Tuncer Hökelek

**Affiliations:** a Department of Chemistry, Zonguldak Karaelmas University, 67100 Zonguldak, Turkey; b Department of Chemistry, Southampton University, Southampton SO17 1BJ, England; c Department of Physics, Hacettepe University, 06800 Beytepe, Ankara, Turkey

## Abstract

In the mol­ecule of the title compound, C_20_H_16_Cl_2_N_2_O_2_, the planar benzimidazole ring system is oriented with respect to the furan and dichloro­benzene rings at dihedral angles of 53.39 (6) and 31.04 (5)°, respectively. In the crystal structure, inter­molecular C—H⋯Cl hydrogen bonds link the mol­ecules into centrosymmetric *R*_2_^2^(8) dimers. These dimers are connected *via* a C—H⋯π contact between the benzimidazole and the furan rings, and π–π contacts between the benz­imidazole and dichloro­benzene ring systems [centroid–centroid distances = 3.505 (1), 3.567 (1), 3.505 (1) and 3.567 (1) Å].

## Related literature

For general background, see: Brammer & Feczko (1988 ▶); Özel Güven *et al.* (2007**a* ▶,b* ▶). For related literature, see: Song & Shin (1998 ▶); Freer *et al.* (1986 ▶); Peeters *et al.* (1996 ▶); Peeters *et al.* (1979**a* ▶,b* ▶); Caira *et al.* (2004 ▶). For ring motif details, see: Bernstein *et al.* (1995 ▶).
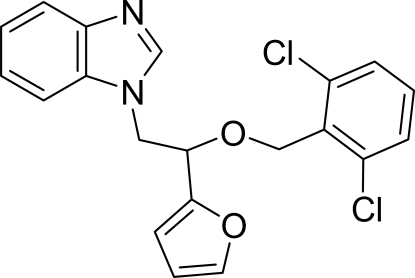


## Experimental

### 

#### Crystal data

C_20_H_16_Cl_2_N_2_O_2_
*M* *_r_* = 387.25
Orthorhombic, 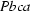

*a* = 12.7720 (3) Å
*b* = 12.9761 (2) Å
*c* = 21.9732 (5) Å
*V* = 3641.63 (13) Å^3^
*Z* = 8
Mo *K*α radiation
μ = 0.37 mm^−1^
*T* = 120 (2) K
0.50 × 0.40 × 0.20 mm

#### Data collection

Bruker Nonius KappaCCD diffractometer
Absorption correction: multi-scan (*SADABS*; Sheldrick, 2007 ▶) *T*_min_ = 0.835, *T*_max_ = 0.929
27000 measured reflections
4181 independent reflections
3311 reflections with *I* > 2σ(*I*)
*R*_int_ = 0.048

#### Refinement

*R*[*F* ^2^ > 2σ(*F* ^2^)] = 0.047
*wR*(*F* ^2^) = 0.122
*S* = 1.10
4181 reflections
300 parameters
All H-atom parameters refined
Δρ_max_ = 0.50 e Å^−3^
Δρ_min_ = −0.49 e Å^−3^

### 

Data collection: *COLLECT* (Hooft, 1998 ▶); cell refinement: *DENZO* (Otwinowski & Minor, 1997 ▶) and *COLLECT*; data reduction: *DENZO* and *COLLECT*; program(s) used to solve structure: *SHELXS97* (Sheldrick, 2008 ▶); program(s) used to refine structure: *SHELXL97* (Sheldrick, 2008 ▶); molecular graphics: *ORTEP-3 for Windows* (Farrugia, 1997 ▶) and *PLATON* (Spek, 2003 ▶); software used to prepare material for publication: *WinGX* (Farrugia, 1999 ▶) and *PLATON*.

## Supplementary Material

Crystal structure: contains datablocks I, global. DOI: 10.1107/S1600536808020758/si2098sup1.cif

Structure factors: contains datablocks I. DOI: 10.1107/S1600536808020758/si2098Isup2.hkl

Additional supplementary materials:  crystallographic information; 3D view; checkCIF report

## Figures and Tables

**Table 1:** Hydrogen-bond geometry (Å, °)

*D*—H⋯*A*	*D*—H	H⋯*A*	*D*⋯*A*	*D*—H⋯*A*
C19—H19⋯Cl2^i^	1.00 (2)	2.76 (2)	3.7470 (19)	172.0 (15)
C1—H1⋯*Cg*1^ii^	0.95 (2)	2.533 (19)	3.441 (2)	158.8 (16)

Symmetry codes: (i) 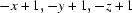; (ii) 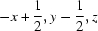.

**Fig. 1.**
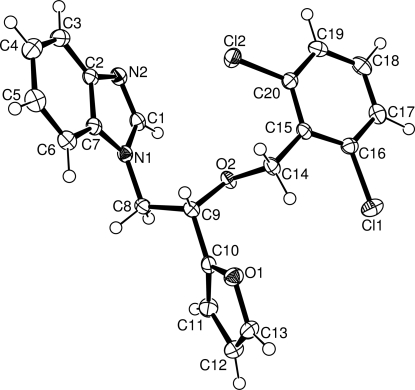
The molecular structure of the title molecule with the atom-numbering scheme. Displacement ellipsoids are drawn at the 50% probability level.

**Fig. 2.**
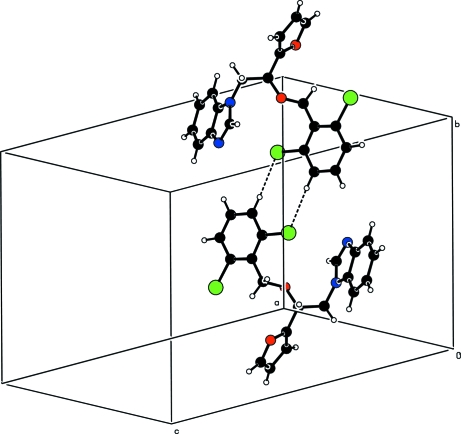
A partial packing diagram of the title compound. Hydrogen bonds are shown as dashed lines.

## supplementary crystallographic information

### Comment

In recent years, there has been increasing interest in synthesis of heterocyclic compounds having biological and commercial importances. Clotrimazole (Song & Shin, 1998), econazole (Freer et al.,  1986), ketoconazole (Peeters et al.,  1979a) and miconazole (Peeters et al.,  1979b) are well-known imidazole ring containing, while itraconazole (Peeters et al.,  1996) and fluconazole (Caira et al.,  2004) are 1H-1,2,4-triazole ring containing, azole derivatives. They have been developed for clinical uses as antifungal agents (Brammer & Feczko, 1988). Lately, similar structures to miconazole and econazole have been reported to show antibacterial activity more than antifungal activity (Özel Güven et al., 2007a,b). In these structures, benzimidazole ring has been found in place of the imidazole ring of miconazole and econazole. We report herein the crystal structure of title benzimidazole derivative.

In the molecule of the title compound (Fig. 1) the bond lengths and angles are generally within normal ranges. The planar benzimidazole ring system is oriented with respect to the furan and dichlorobenzene rings at dihedral angles of 53.39 (6)° and 31.04 (5)°, respectively. Atoms C8, C9 and C14 are 0.063 (2), 0.065 (2) and -0.039 (2) Å away from the ring planes of benzimidazole, furan and dichlorobenzene, respectively. So, they are coplanar with the adjacent rings. The N1-C8-C9, C9-O2-C14 and C8-C9-C10, O2-C9-C10 bond angles are nearly equal, while O2-C9-C8 and O2-C14-C15 bond angles are different from each other. The N1-C1-N2, N2-C2-C7 and C2-C7-C6 bond angles are enlarged, while C5-C6-C7 bond angle is narrowed. In dichlorobenzene ring, the C15-C16-C17 and C15-C20-C19 bond angles are enlarged, while C16-C15-C20 bond angle is highly narrowed (Table 1), probably due to the intermolecular C-H···Cl hydrogen bonds (Table 2).

In the crystal structure, intermolecular weak C-H···Cl hydrogen bonds (Table 2) link the molecules to form a R^2^_2_(8) ring motif (Fig. 2) (Bernstein et al.,  1995), in which they may be effective in the stabilization of the structure. The C—H···π contact (Table 2) between the benzimidazole and the furan rings and π—π contacts between the benzimidazole and dichlorobenzene ring systems Cg2···Cg4^i^, Cg3···Cg4^i^, Cg4···Cg2^ii^ and Cg4···Cg3^ii^ [symmetry codes: (i) -1/2 + x, 1/2 - y, 1 - z; (ii) 1/2 + x, 1/2 - y, 1 - z, where Cg2, Cg3 and Cg4 are centroids of the rings (N1/N2/C1/C2/C7), (C2-C7) and (C15-C20), respectively] further stabilize the structure, with centroid–centroid distances of 3.505 (1), 3.567 (1), 3.505 (1) and 3.567 (1) Å, respectively.

### Experimental

The title compound, was synthesized by the reaction of 2-(1*H*-benzimidazol -1-yl)-1-(furan-2-yl)ethanol (Özel Güven *et al.*, 2007*b*) with NaH and appropriate benzyl halide. A solution of alcohol (150 mg, 0.657 mmol) in DMF (1.5 ml) was added to NaH (19.7 mg, 0.821 mmol) in small fractions. The appropriate benzyl halide (158 mg, 0.657 mmol) in DMF (0.8 ml) was then added dropwise. The mixture was stirred at room temperature for 2 h, and the excess hydride was decomposed with a small amount of methyl alcohol. After evaporation to dryness under reduced pressure, the crude residue was suspended with water and extracted with methylene chloride. The organic layer was dried over anhydrous sodium sulfate, and then evaporated to dryness. The crude residue was purified by chromatography on a silica-gel column using chloroform-methanol as eluent. Crystals suitable for X-ray analysis were obtained by the recrystallization of the ether from a mixture of hexane/ethyl acetate (1:2) (yield; 124 mg, 49%).

### Refinement

H atoms were located in difference syntheses and refined isotropically [C—H = 0.92 (2)–1.03 (2) Å; *U*_iso_(H) = 0.015 (4)–0.037 (6) Å^2^].

### Crystal data

**Table:**

C_20_H_16_Cl_2_N_2_O_2_	*F*_000_ = 1600
*M**_r_* = 387.25	*D*_x_ = 1.413 Mg m^−^^3^
Orthorhombic, *P**b**c**a*	Mo *K*α radiation λ = 0.71073 Å
Hall symbol: -P 2ac 2ab	Cell parameters from 4594 reflections
*a* = 12.7720 (3) Å	θ = 2.9–27.5º
*b* = 12.9761 (2) Å	µ = 0.37 mm^−^^1^
*c* = 21.9732 (5) Å	*T* = 120 (2) K
*V* = 3641.63 (13) Å^3^	Block, colorless
*Z* = 8	0.50 × 0.40 × 0.20 mm

### Data collection

**Table:**

Bruker Nonius KappaCCD diffractometer	4181 independent reflections
Radiation source: fine-focus sealed tube	3311 reflections with *I* > 2σ(*I*)
Monochromator: graphite	*R*_int_ = 0.048
Detector resolution: 9.091 pixels mm^-1^	θ_max_ = 27.8º
*T* = 120(2) K	θ_min_ = 3.1º
φ and ω scans	*h* = −13→16
Absorption correction: multi-scan(SADABS; Sheldrick, 2007)	*k* = −14→16
*T*_min_ = 0.835, *T*_max_ = 0.929	*l* = −28→22
27000 measured reflections	

### Refinement

**Table:**

Refinement on *F*^2^	Hydrogen site location: inferred from neighbouring sites
Least-squares matrix: full	All H-atom parameters refined
*R*[*F*^2^ > 2σ(*F*^2^)] = 0.047	*w* = 1/[σ^2^(*F*_o_^2^) + (0.0688*P*)^2^ + 0.7891*P*] where *P* = (*F*_o_^2^ + 2*F*_c_^2^)/3
*wR*(*F*^2^) = 0.122	(Δ/σ)_max_ < 0.001
*S* = 1.10	Δρ_max_ = 0.50 e Å^−^^3^
4181 reflections	Δρ_min_ = −0.49 e Å^−^^3^
300 parameters	Extinction correction: SHELXL97 (Sheldrick, 2008), Fc^*^=kFc[1+0.001xFc^2^λ^3^/sin(2θ)]^-1/4^
Primary atom site location: structure-invariant direct methods	Extinction coefficient: 0.0141 (10)
Secondary atom site location: difference Fourier map	

### Special details

**Table:**

Geometry. All e.s.d.'s (except the e.s.d. in the dihedral angle between two l.s. planes) are estimated using the full covariance matrix. The cell e.s.d.'s are taken into account individually in the estimation of e.s.d.'s in distances, angles and torsion angles; correlations between e.s.d.'s in cell parameters are only used when they are defined by crystal symmetry. An approximate (isotropic) treatment of cell e.s.d.'s is used for estimating e.s.d.'s involving l.s. planes.
Refinement. Refinement of *F*^2^ against ALL reflections. The weighted *R*-factor *wR* and goodness of fit *S* are based on *F*^2^, conventional *R*-factors *R* are based on *F*, with *F* set to zero for negative *F*^2^. The threshold expression of *F*^2^ > σ(*F*^2^) is used only for calculating *R*-factors(gt) *etc*. and is not relevant to the choice of reflections for refinement. *R*-factors based on *F*^2^ are statistically about twice as large as those based on *F*, and *R*- factors based on ALL data will be even larger.

### Fractional atomic coordinates and isotropic or equivalent isotropic displacement parameters (Å^2^)

**Table:**

	*x*	*y*	*z*	*U*_iso_*/*U*_eq_	
Cl1	0.52705 (4)	0.15868 (4)	0.27568 (2)	0.03062 (16)	
Cl2	0.46363 (3)	0.33374 (4)	0.49707 (2)	0.02746 (16)	
O1	0.44665 (9)	−0.10157 (10)	0.42074 (6)	0.0231 (3)	
O2	0.36998 (8)	0.13809 (9)	0.42664 (5)	0.0192 (3)	
N1	0.24117 (10)	0.14633 (11)	0.53080 (7)	0.0183 (3)	
N2	0.22763 (11)	0.31228 (12)	0.56340 (7)	0.0216 (3)	
C1	0.21768 (13)	0.24739 (13)	0.51763 (9)	0.0205 (4)	
H1	0.1946 (14)	0.2656 (15)	0.4777 (9)	0.018 (5)*	
C2	0.26218 (12)	0.24900 (13)	0.61103 (8)	0.0186 (4)	
C3	0.28517 (13)	0.27431 (15)	0.67109 (9)	0.0241 (4)	
H3	0.2801 (14)	0.3425 (15)	0.6832 (9)	0.018 (5)*	
C4	0.31590 (13)	0.19444 (16)	0.70928 (10)	0.0270 (4)	
H4	0.3323 (15)	0.2095 (16)	0.7491 (10)	0.026 (5)*	
C5	0.32404 (14)	0.09079 (16)	0.68881 (9)	0.0269 (4)	
H5	0.3477 (15)	0.0361 (15)	0.7160 (9)	0.026 (5)*	
C6	0.30207 (13)	0.06387 (14)	0.62937 (9)	0.0223 (4)	
H6	0.3068 (15)	−0.0068 (17)	0.6154 (9)	0.028 (5)*	
C7	0.27122 (12)	0.14474 (13)	0.59120 (8)	0.0186 (4)	
C8	0.24218 (13)	0.05901 (14)	0.48853 (9)	0.0198 (4)	
H81	0.1896 (15)	0.0733 (16)	0.4578 (9)	0.026 (5)*	
H82	0.2223 (14)	−0.0048 (16)	0.5104 (8)	0.020 (5)*	
C9	0.34849 (12)	0.04456 (13)	0.46028 (8)	0.0178 (4)	
H9	0.3996 (14)	0.0367 (14)	0.4940 (8)	0.015 (4)*	
C10	0.35358 (12)	−0.05012 (13)	0.42090 (8)	0.0190 (4)	
C11	0.28836 (14)	−0.09792 (15)	0.38168 (9)	0.0249 (4)	
H11	0.2189 (17)	−0.0773 (17)	0.3727 (10)	0.035 (6)*	
C12	0.34340 (15)	−0.18372 (15)	0.35581 (9)	0.0266 (4)	
H12	0.3178 (16)	−0.2308 (17)	0.3271 (10)	0.031 (6)*	
C13	0.43807 (15)	−0.18196 (15)	0.38007 (9)	0.0252 (4)	
H13	0.4966 (17)	−0.2303 (18)	0.3736 (9)	0.032 (6)*	
C14	0.47740 (13)	0.14762 (14)	0.41337 (9)	0.0206 (4)	
H141	0.5151 (15)	0.1398 (14)	0.4508 (10)	0.019 (5)*	
H142	0.4958 (16)	0.0907 (17)	0.3863 (10)	0.030 (5)*	
C15	0.49591 (12)	0.25242 (13)	0.38536 (8)	0.0177 (4)	
C16	0.51955 (13)	0.26642 (14)	0.32382 (8)	0.0204 (4)	
C17	0.53897 (13)	0.36388 (15)	0.29854 (9)	0.0243 (4)	
H17	0.5544 (16)	0.3651 (17)	0.2524 (11)	0.037 (6)*	
C18	0.53539 (14)	0.45094 (15)	0.33540 (10)	0.0263 (4)	
H18	0.5515 (16)	0.5199 (17)	0.3202 (10)	0.031 (6)*	
C19	0.51163 (13)	0.44195 (14)	0.39651 (9)	0.0232 (4)	
H19	0.5110 (15)	0.5037 (16)	0.4233 (9)	0.025 (5)*	
C20	0.49238 (13)	0.34367 (14)	0.42002 (8)	0.0190 (4)	

### Atomic displacement parameters (Å^2^)

**Table:**

	*U*^11^	*U*^22^	*U*^33^	*U*^12^	*U*^13^	*U*^23^
Cl1	0.0346 (3)	0.0290 (3)	0.0282 (3)	0.00275 (18)	−0.0013 (2)	−0.0095 (2)
Cl2	0.0261 (2)	0.0342 (3)	0.0221 (3)	−0.00255 (17)	0.00426 (17)	−0.00402 (19)
O1	0.0216 (6)	0.0225 (7)	0.0253 (7)	0.0020 (5)	0.0008 (5)	−0.0037 (5)
O2	0.0150 (5)	0.0167 (6)	0.0258 (7)	−0.0010 (4)	0.0025 (5)	0.0039 (5)
N1	0.0166 (7)	0.0175 (8)	0.0208 (8)	0.0010 (5)	0.0026 (6)	0.0011 (6)
N2	0.0190 (7)	0.0190 (8)	0.0268 (9)	0.0019 (6)	0.0037 (6)	0.0013 (6)
C1	0.0179 (8)	0.0191 (9)	0.0246 (10)	0.0015 (7)	0.0028 (7)	0.0036 (8)
C2	0.0132 (7)	0.0181 (9)	0.0246 (10)	0.0007 (6)	0.0035 (7)	−0.0012 (7)
C3	0.0175 (8)	0.0240 (10)	0.0308 (11)	0.0001 (7)	0.0023 (7)	−0.0072 (8)
C4	0.0187 (8)	0.0375 (11)	0.0249 (11)	0.0014 (7)	−0.0022 (7)	−0.0044 (9)
C5	0.0209 (8)	0.0288 (10)	0.0309 (11)	0.0035 (7)	−0.0013 (8)	0.0042 (9)
C6	0.0189 (8)	0.0202 (9)	0.0279 (10)	0.0018 (7)	0.0008 (7)	0.0019 (8)
C7	0.0125 (7)	0.0208 (9)	0.0224 (9)	0.0002 (6)	0.0021 (7)	−0.0005 (7)
C8	0.0180 (8)	0.0184 (9)	0.0228 (10)	−0.0029 (6)	0.0009 (7)	−0.0012 (7)
C9	0.0175 (8)	0.0162 (8)	0.0198 (9)	−0.0010 (6)	−0.0004 (7)	0.0017 (7)
C10	0.0189 (8)	0.0187 (9)	0.0194 (9)	0.0001 (6)	0.0034 (7)	0.0029 (7)
C11	0.0235 (9)	0.0273 (10)	0.0240 (10)	−0.0006 (7)	−0.0010 (7)	−0.0003 (8)
C12	0.0321 (10)	0.0253 (10)	0.0224 (10)	−0.0033 (8)	0.0019 (8)	−0.0047 (8)
C13	0.0304 (9)	0.0216 (9)	0.0235 (10)	0.0019 (7)	0.0060 (8)	−0.0044 (8)
C14	0.0154 (8)	0.0179 (9)	0.0285 (11)	−0.0003 (6)	0.0027 (7)	0.0026 (8)
C15	0.0118 (7)	0.0182 (9)	0.0233 (9)	0.0002 (6)	0.0006 (7)	0.0012 (7)
C16	0.0166 (8)	0.0214 (9)	0.0232 (10)	0.0002 (6)	−0.0015 (7)	−0.0027 (7)
C17	0.0214 (9)	0.0286 (10)	0.0229 (11)	−0.0012 (7)	−0.0019 (7)	0.0061 (8)
C18	0.0243 (9)	0.0203 (10)	0.0344 (11)	−0.0028 (7)	−0.0040 (8)	0.0076 (8)
C19	0.0198 (8)	0.0188 (9)	0.0311 (11)	−0.0009 (7)	−0.0033 (7)	−0.0026 (8)
C20	0.0144 (7)	0.0225 (9)	0.0200 (9)	0.0006 (6)	−0.0009 (7)	−0.0012 (7)

### Geometric parameters (Å, °)

**Table:**

Cl1—C16	1.7557 (19)	C8—H82	0.99 (2)
Cl2—C20	1.7373 (19)	C9—C8	1.505 (2)
O1—C10	1.363 (2)	C9—C10	1.504 (2)
O1—C13	1.378 (2)	C9—H9	0.992 (18)
O2—C9	1.4474 (19)	C10—C11	1.350 (2)
O2—C14	1.4081 (19)	C11—C12	1.434 (3)
N1—C1	1.376 (2)	C11—H11	0.95 (2)
N1—C7	1.382 (2)	C12—H12	0.94 (2)
N1—C8	1.465 (2)	C13—C12	1.322 (3)
N2—C1	1.318 (2)	C13—H13	0.99 (2)
C1—H1	0.95 (2)	C14—H141	0.96 (2)
C2—N2	1.401 (2)	C14—H142	0.98 (2)
C2—C3	1.391 (3)	C15—C14	1.511 (2)
C2—C7	1.426 (2)	C15—C16	1.397 (3)
C3—H3	0.926 (19)	C15—C20	1.409 (2)
C4—C3	1.390 (3)	C17—C16	1.403 (3)
C4—C5	1.422 (3)	C17—C18	1.391 (3)
C4—H4	0.92 (2)	C17—H17	1.03 (2)
C5—H5	0.98 (2)	C18—H18	0.98 (2)
C6—C5	1.381 (3)	C19—C18	1.382 (3)
C6—C7	1.400 (2)	C19—H19	0.99 (2)
C6—H6	0.97 (2)	C20—C19	1.398 (2)
C8—H81	0.97 (2)		
			
C10—O1—C13	107.64 (14)	C10—C9—H9	108.5 (10)
C14—O2—C9	111.36 (12)	O1—C10—C9	116.05 (14)
C1—N1—C7	106.07 (15)	C11—C10—O1	108.15 (15)
C1—N1—C8	127.26 (16)	C11—C10—C9	135.73 (15)
C7—N1—C8	126.52 (15)	C10—C11—C12	107.90 (16)
C1—N2—C2	103.05 (15)	C10—C11—H11	125.6 (14)
N1—C1—H1	119.8 (12)	C12—C11—H11	126.5 (14)
N2—C1—N1	115.30 (17)	C11—C12—H12	127.0 (13)
N2—C1—H1	124.9 (12)	C13—C12—C11	105.97 (18)
N2—C2—C7	110.68 (15)	C13—C12—H12	127.0 (13)
C3—C2—N2	129.52 (17)	O1—C13—H13	121.0 (12)
C3—C2—C7	119.78 (16)	C12—C13—O1	110.33 (17)
C4—C3—C2	117.14 (18)	C12—C13—H13	128.6 (12)
C4—C3—H3	123.9 (12)	O2—C14—C15	108.41 (13)
C2—C3—H3	118.9 (12)	O2—C14—H141	107.6 (12)
C3—C4—C5	122.33 (19)	O2—C14—H142	107.1 (12)
C3—C4—H4	118.7 (13)	C15—C14—H141	111.5 (12)
C5—C4—H4	119.0 (13)	C15—C14—H142	113.3 (13)
C4—C5—H5	121.1 (12)	H141—C14—H142	108.7 (17)
C6—C5—C4	121.58 (18)	C16—C15—C20	114.88 (15)
C6—C5—H5	117.3 (12)	C16—C15—C14	123.02 (16)
C5—C6—C7	115.74 (17)	C20—C15—C14	122.09 (16)
C5—C6—H6	121.8 (12)	C15—C16—C17	122.57 (16)
C7—C6—H6	122.5 (12)	C15—C16—Cl1	119.42 (13)
N1—C7—C6	131.67 (17)	C17—C16—Cl1	118.00 (14)
N1—C7—C2	104.89 (14)	C16—C17—H17	115.9 (12)
C6—C7—C2	123.42 (17)	C18—C17—C16	119.71 (18)
N1—C8—C9	111.46 (14)	C18—C17—H17	124.4 (12)
N1—C8—H81	106.7 (12)	C17—C18—H18	122.6 (13)
N1—C8—H82	109.6 (11)	C19—C18—C17	120.31 (18)
C9—C8—H81	111.2 (12)	C19—C18—H18	117.1 (13)
C9—C8—H82	109.1 (11)	C18—C19—C20	118.35 (18)
H81—C8—H82	108.7 (16)	C18—C19—H19	120.6 (12)
O2—C9—C10	112.52 (14)	C20—C19—H19	121.0 (12)
O2—C9—C8	106.10 (13)	C15—C20—Cl2	118.12 (13)
O2—C9—H9	110.0 (10)	C19—C20—C15	124.17 (17)
C8—C9—H9	107.4 (10)	C19—C20—Cl2	117.71 (14)
C10—C9—C8	112.22 (14)		
			
C13—O1—C10—C11	0.53 (19)	C5—C6—C7—C2	−0.1 (2)
C13—O1—C10—C9	−176.90 (14)	O2—C9—C8—N1	−61.67 (18)
C10—O1—C13—C12	−1.1 (2)	O2—C9—C10—C11	−81.3 (2)
C14—O2—C9—C10	−74.57 (17)	O2—C9—C10—O1	95.24 (17)
C14—O2—C9—C8	162.36 (15)	C8—C9—C10—C11	38.3 (3)
C9—O2—C14—C15	−173.07 (14)	C8—C9—C10—O1	−145.19 (15)
C7—N1—C1—N2	−0.99 (19)	C10—C9—C8—N1	175.07 (14)
C8—N1—C1—N2	−176.72 (15)	O1—C10—C11—C12	0.1 (2)
C1—N1—C7—C2	0.65 (16)	C9—C10—C11—C12	176.83 (18)
C1—N1—C7—C6	178.95 (17)	C10—C11—C12—C13	−0.8 (2)
C8—N1—C7—C2	176.41 (14)	O1—C13—C12—C11	1.1 (2)
C8—N1—C7—C6	−5.3 (3)	C16—C15—C14—O2	−108.33 (18)
C1—N1—C8—C9	90.6 (2)	C20—C15—C14—O2	72.8 (2)
C7—N1—C8—C9	−84.3 (2)	C14—C15—C16—Cl1	0.8 (2)
C2—N2—C1—N1	0.85 (18)	C14—C15—C16—C17	−178.50 (15)
C3—C2—N2—C1	−179.27 (17)	C20—C15—C16—Cl1	179.66 (11)
C7—C2—N2—C1	−0.38 (17)	C20—C15—C16—C17	0.4 (2)
N2—C2—C3—C4	178.50 (16)	C14—C15—C20—Cl2	−1.0 (2)
C7—C2—C3—C4	−0.3 (2)	C14—C15—C20—C19	178.26 (15)
N2—C2—C7—N1	−0.18 (17)	C16—C15—C20—Cl2	−179.96 (12)
N2—C2—C7—C6	−178.66 (15)	C16—C15—C20—C19	−0.7 (2)
C3—C2—C7—N1	178.83 (14)	C18—C17—C16—Cl1	−178.99 (13)
C3—C2—C7—C6	0.3 (2)	C18—C17—C16—C15	0.3 (3)
C5—C4—C3—C2	0.0 (3)	C16—C17—C18—C19	−0.8 (3)
C3—C4—C5—C6	0.3 (3)	C20—C19—C18—C17	0.5 (3)
C7—C6—C5—C4	−0.2 (2)	Cl2—C20—C19—C18	179.51 (13)
C5—C6—C7—N1	−178.12 (16)	C15—C20—C19—C18	0.2 (3)

### Hydrogen-bond geometry (Å, °)

**Table:**

*D*—H···*A*	*D*—H	H···*A*	*D*···*A*	*D*—H···*A*
C19—H19···Cl2^i^	1.00 (2)	2.76 (2)	3.7470 (19)	172.0 (15)
C1—H1···Cg1^ii^	0.95 (2)	2.533 (19)	3.441 (2)	158.8 (16)

Symmetry codes: (i) −*x*+1, −*y*+1, −*z*+1; (ii) −*x*+1/2, *y*−1/2, *z*.
